# Dynamics of Mycotoxins, Microbial Communities, and Quality of Corn When Stored at Various Temperatures

**DOI:** 10.3390/foods15193438

**Published:** 2026-09-25

**Authors:** Yixuan Yuan, Pengwen Lyu, Ze Shi, Junze Li, Xuenan Li, Dan Cai

**Affiliations:** 1 College of Food Science and Engineering, Jilin Agricultural University, Changchun 130118, China; yyx2024sj@163.com (Y.Y.); lvpengwen@163.com (P.L.); 15114470377@163.com (Z.S.); ljz66608@outlook.com (J.L.); 2 National Engineering Laboratory for Wheat and Corn Deep Processing, Changchun 130118, China

**Keywords:** corn, long-term storage, mycotoxins, fungal community, quality deterioration, storage temperature

## Abstract

Corn is a major grain crop in China; however, mycotoxin contamination during storage threatens grain quality. In this study, naturally contaminated corn was stored at 15 °C or 35 °C under 65 ± 5% relative humidity for 8 months, with monthly sampling. Mycotoxin contents and fungal communities were analyzed, and the major quality indicators of corn were evaluated. Under both storage conditions, aflatoxins and ochratoxin A remained below detection limits. At 15 °C, fumonisins B_1_ and B_2_ decreased by 3.2% and 2.5%, respectively, after 8 months of storage. FB_3_ reached 41.51 ± 2.10 μg/kg at month 8, representing a 17.3% decrease. Deoxynivalenol and zearalenone remained relatively stable. At 35 °C, the concentrations of FB_1_, FB_2_, and FB_3_ reached 414.85 ± 3.27, 59.52 ± 1.53, and 91.76 ± 2.04 μg/kg, respectively. These five toxins increased by 13.8%, 40.0%, 82.8%, 10.4%, and 26.1%, respectively. Analysis of fungal communities indicated that dominant taxa differed between storage conditions, with higher relative abundance of *Fusarium* at 35 °C and higher *Trichoderma* at 15 °C. Storage at 35 °C resulted in a significant decrease in crude fat, starch content, germination rate, and antioxidant enzyme activity compared with conditions at 15 °C, while free fatty acid content increased. Storage at 15 °C limited toxin accumulation and quality deterioration compared with 35 °C. Temperature control can therefore improve corn preservation.

## 1. Introduction

Corn (*Zea mays* L.) is an important cereal crop worldwide [1]. Storage is a critical stage for post-harvest loss and mycotoxin contamination in corn. Previous studies have examined the occurrence of mycotoxins in food crops worldwide [2]. Understanding how mycotoxin concentrations change during storage, particularly under different temperature conditions, is therefore important for managing contamination and preserving corn quality.

During storage, corn is susceptible to infection by various fungi, including *Aspergillus*, *Fusarium*, and *Penicillium* [3,4], which can produce a range of highly toxic mycotoxins. Aflatoxins (AFs), fumonisins (FBs), zearalenone (ZEN), deoxynivalenol (DON), and ochratoxin A (OTA) are of particular concern owing to their high toxicity and frequent occurrence in corn [5,6]. These mycotoxins exhibit distinct target-organ toxicities, including hepatic and renal damage, reproductive and immune suppression, genotoxicity, and potential carcinogenicity [7]. Their frequent co-occurrence in corn may lead to synergistic toxic effects, and their transfer through the food chain poses long-term risks to livestock production and human health [8].

Storage temperature is a key environmental factor that regulates mycotoxin dynamics during corn storage [9]. Changes in mycotoxin levels under different temperature conditions are highly variable because individual mycotoxins differ in chemical stability, and toxigenic fungi differ in temperature adaptability. Low temperatures generally slow spore germination, hyphal extension, and the expression of genes involved in mycotoxin biosynthesis. In contrast, elevated temperatures may favor the growth of toxigenic fungi and toxin production by promoting the activation of toxin biosynthetic genes. Temperature conditions favoring fungal growth may differ from those favoring mycotoxin production. For example, Yu et al. [10] described approximately 30 °C as optimal for aflatoxin biosynthesis and approximately 37 °C as optimal for *Aspergillus flavus* growth. Their RNA-Seq analysis showed substantially higher expression of aflatoxin biosynthesis genes at 30 °C than at 37 °C, indicating that a higher growth temperature does not necessarily favor toxin biosynthesis.

Mycotoxin contamination and quality deterioration during corn storage are interrelated. Fungal metabolism and toxin accumulation can further damage the kernel structure and impair physiological activity [11,12]. Studies indicated that if stored improperly, corn seeds could become contaminated with mycotoxins, which could affect seed germination rates, enzyme activity, and other physiological indicators [13,14]. Consequently, the degradation of nutritional components, increase in free fatty acids, decline in germination rate, and loss of antioxidant enzyme activities may create favorable conditions for toxigenic fungal infection and toxin biosynthesis. However, further evidence is needed on how the relationships among fungal community succession, mycotoxin accumulation, and quality deterioration vary with storage temperature in naturally contaminated corn.

Topan et al. evaluated the quality of corn stored for 5 months in a 2500 t silo at 13 °C [15]. Rodrigues et al. monitored the quality of stored corn kernels at 30–36 °C and a relative humidity close to 70% [16]. Chen et al. monitored corn stored for one year in large commercial warehouses; when an inner cyclic cooling aeration system was used, the relative humidity remained below 65%. Without the inner cyclic cooling aeration system, the relative humidity in the headspace of the stored corn typically exceeded 75% [17]. Therefore, to investigate the effects of different temperatures on mycotoxins during corn storage, two temperature conditions (15 °C and 35 °C) were selected to represent low and high temperatures, respectively. Under constant relative humidity (65 ± 5%), the changes in corn quality during 8 months of long-term storage were investigated.

Mycotoxins were quantified using HPLC–MS/MS, and fungal communities were characterized by ITS1 amplicon sequencing. Nutritional and physiological indicators were measured to examine their associations with mycotoxin concentrations within each temperature condition. These results provide a reference for monitoring mycotoxin risk and improving quality and safety controls during long-term corn storage.

## 2. Materials and Methods

### 2.1. Materials and Reagents

A total of 100 kg of corn (Meiya 81) was collected from field-grown plants in Songyuan, Jilin Province, China. The corn was harvested at physiological maturity in 2024. Prior to the start of the storage trial, the corn kernels were threshed and placed in a 101A-3ET electric hot-air dryer (Shanghai Laboratory Instrument Factory Co., Ltd., Shanghai, China), where they were dried with hot air at 60 °C for 4 h; the drying process was terminated when the moisture content of the kernels dropped to 14.5%. Mixed standard solutions of AFB_1_, AFB_2_, AFG_1_, and AFG_2_ (100 μg/mL each) and FB_1_, FB_2_, and FB_3_ (100 μg/mL each) were purchased from Alta Scientific Co., Ltd. (Tianjin, China). OTA, DON, and ZEN individual standard solutions (100 μg/mL each) were purchased from NCS Testing Technology Co., Ltd. (Beijing, China). HPLC-grade acetonitrile and methanol were purchased from Sigma-Aldrich (St. Louis, MO, USA). HPLC-grade glacial acetic acid was obtained from Shanghai Macklin Biochemical Co., Ltd. (Shanghai, China).

Absolute ethanol, potassium hydroxide, sodium hydroxide, hydrochloric acid, concentrated sulfuric acid, copper sulfate, potassium sulfate, phenolphthalein indicator, anthrone, diethyl ether, and petroleum ether were purchased from Sinopharm Chemical Reagent Co., Ltd. (Shanghai, China). A glucose standard (purity ≥ 99.5%) was obtained from Shanghai Yuanye Bio-Technology Co., Ltd. (Shanghai, China). Phosphate-buffered saline (PBS), the total superoxide dismutase (SOD) assay kit (BC0170), and the catalase (CAT) assay kit (BC0200) were purchased from Beijing Solarbio Science and Technology Co., Ltd. (Beijing, China). The MagBeads FastDNA Kit for Soil (116564384) was obtained from MP Biomedicals (Santa Ana, CA, USA). Agarose (75510-019) and TAE buffer (AM9870) were obtained from Invitrogen (Carlsbad, CA, USA). Q5 High-Fidelity DNA Polymerase (M0491L) was obtained from New England Biolabs (Beijing) Ltd. (NEB, Beijing, China).

### 2.2. Long-Term Storage Experiment

The 100 kg of corn samples were thoroughly mixed using a mixer, then weighed into six equal portions of 10 kg each and placed into breathable nylon mesh bags. The six independent constant-temperature and constant-humidity incubators (Ningbo Dongnan Instrument Co., Ltd., Ningbo, China) were randomly assigned to either the 15 °C or 35 °C group, with three incubators in each group. The corn was stored for eight months, with relative humidity set at 65 ± 5%. Temperature and relative humidity readings inside the chambers were continuously monitored, maintaining a temperature control tolerance of ±2 °C. Samples were collected once a month throughout the storage period. During each sampling, 10 samples, each weighing 100 g, were collected from different locations within each bag (including the top, middle, and bottom layers, as well as both sides). These incremental samples were combined into a composite sample weighing 1 kg for analysis.

### 2.3. Mycotoxin Extraction and HPLC–MS/MS Analysis

#### 2.3.1. Sample Preparation

After grinding the mixed corn in a high-speed grinder (Yongkang Hongtai Yang Mechanical & Electrical Co., Ltd., Zhejiang, China), a sample of corn flour (10.00 ± 0.05 g) was extracted with 40 mL of 79% acetonitrile containing 1% acetic acid and stirred for 30 min by vortex mixing. The extract was centrifuged at 4000 r/min for 10 min, and the supernatant (0.5 mL) was mixed with an equal volume of the extraction solvent. After centrifugation at 12,000 r/min for 15 min at 4 °C, the supernatant was passed through a 0.22 μm nylon membrane filter before analysis.

#### 2.3.2. Chromatographic and Mass Spectrometric Conditions

Chromatographic separation was performed using a Waters ACQUITY H-Class liquid chromatography system (Waters, Milford, MA, USA) equipped with a Phenomenex F5 column (4.6 mm × 100 mm, 2.6 μm). The column temperature was maintained at 25 °C, and the injection volume was 10 μL. Mobile phase A was 0.1% (*v*/*v*) formic acid in water, and mobile phase B was acetonitrile. The flow rate was set to 0.3 mL/min. The specific gradient elution parameters are provided in the Supplementary Materials.

Mass spectrometry was performed using an AB Sciex 4500 triple-quadrupole mass spectrometer equipped with an electrospray ionization (ESI) source (AB Sciex, Framingham, MA, USA). Data were acquired in multiple reaction monitoring (MRM) mode under both positive and negative ionization modes. In the positive ion mode, the ion source temperature and ion spray voltage were set at 550 °C and 5500 V, respectively, whereas in the negative ion mode, they were set at 600 °C and −4500 V, respectively. The curtain gas was set at 25 psi for both ionization modes. The nebulizer gas was set at 55 and 60 psi, and the auxiliary heating gas was set at 55 and 50 psi in the positive and negative ion modes, respectively. The collision gas was set to 6/Medium. The quantitative ions, qualitative ions, collision energies, declustering potential, and retention times for each mycotoxin are presented in the Supplementary Materials. This liquid-phase method can be used to detect mycotoxins in corn; the method validation data are described in our previous report [18].

### 2.4. Fungal Community Sequencing and Bioinformatics Analysis

DNA extraction and fungal community analysis were performed using milled and homogenized corn samples. At each sampling time, corn kernels were collected from multiple positions within each storage bag and combined to form a composite sample for that bag. The material was ground to pass through a 1 mm sieve, with retained material reground and incorporated into the sample. Briefly, 1 g of well-mixed corn sample was collected under aseptic conditions, rapidly frozen in liquid nitrogen, and stored at −80 °C. Before DNA extraction, a 0.2–0.5 g portion of each stored sample was homogenized in extraction lysis buffer using a Tissuelyser-48 tissue grinder (Shanghai Jingxin, Shanghai, China) at 60 Hz. Total genomic DNA samples were extracted using the MagBeads FastDNA Kit for Soil (116564384), following the manufacturer’s instructions, and stored at −20 °C prior to further analysis. DNA integrity was assessed by 0.8% agarose gel electrophoresis, and DNA concentration and purity were assessed using a NanoDrop NC2000 spectrophotometer (Thermo Fisher Scientific, Waltham, MA, USA).

The ITS1 region of fungi was targeted with primers ITS1F (5’-barcode+CTTGGTCATTTAGAGGAAGTAA-3’) and LR3 (5’-CCGTGTTTCAAGACGGG-3’). The PCR conditions for amplifying the ITS fragments consisted of an initial denaturation at 98 °C for 5 min; 30 cycles of denaturation at 98 °C for 30 s, annealing at 56 °C for 30 s, and extension at 72 °C for 45 s; and a final extension at 72 °C for 10 min. Then, the amplicons were sequenced using the Illumina MiSeq platform. The sequencing depth was assessed using rarefaction curves and Good’s coverage. Alpha diversity was evaluated using the Chao1, observed species, and Shannon, Simpson, and Pielou’s evenness indices to compare the fungal community richness, diversity, and evenness under different storage conditions. Principal coordinate analysis (PCoA) based on Bray–Curtis distances was performed to examine the differences in fungal community structure among the groups. Fungal community composition was further analyzed at the phylum, genus, and species levels to investigate the succession of the dominant fungal taxa during storage at different temperatures.

### 2.5. Measurement of Corn Quality Indicators

Corn quality indicators were measured separately for each of the three independent storage replicates per temperature at each sampling time. Samples from different storage replicates were not pooled, and one result per indicator was obtained for each storage replicate.

#### 2.5.1. Measurement of Corn Nutritional Quality

##### Crude Protein Content

The crude protein content was determined using the Kjeldahl method according to the Chinese National Standard GB 5009.5-2025 [19]. Corn flour (0.500 g) was digested with copper sulfate, potassium sulfate, and concentrated sulfuric acid until the digest became clear. The digest was distilled and titrated with a standard hydrochloric acid solution, and the crude protein content was then calculated. A reagent blank was also prepared in parallel.

##### Crude Fat Content

Crude fat content was determined using the acid hydrolysis method according to the Chinese national standard GB 5009.6-2025 [20]. Corn flour (2.000 g) was hydrolyzed with hydrochloric acid, and the released crude fat was extracted repeatedly with diethyl ether. The extracts were combined, and the solvent was removed. The receiving flask was dried to a constant weight, and the crude fat content was calculated from the mass of the residue.

##### Starch Content

Starch content was estimated using a modified acid hydrolysis procedure based on GB 5009.9-2023 [21]. Corn flour (1.000 g) was defatted with diethyl ether, hydrolyzed with hydrochloric acid, and neutralized with sodium hydroxide. The hydrolysate was diluted to a fixed volume and filtered, and the filtrate was collected for analysis. Briefly, 0.0, 0.2, 0.4, 0.6, 0.8, and 1.0 mL of a 1 mg/mL glucose standard solution were pipetted into separate colorimetric tubes and diluted with water to a total volume of 2 mL. Then, 6 mL of anthrone reagent was added and shaken to mix. The mixture was heated in a boiling water bath for 10 min and then cooled to room temperature. Absorbance was measured at a wavelength of 620 nm. A standard curve was plotted with glucose content on the *x*-axis and absorbance on the *y*-axis, and a regression equation was derived. The starch content was calculated from the standard curve.

(1) $$X=\frac{q\times V\times 0.9}{m\times v}$$

where *X* is starch content (mg/g); *q* is the glucose equivalents derived from the calibration curve (mg); *V* is the total hydrolysate volume (mL); and *m* is the sample mass (g). *v* is the volume of the liquid to be tested (mL). The factor 0.9 converts glucose mass to starch mass.

##### Free Fatty Acids

The free fatty acids were determined according to the Chinese National Standard GB/T 20570-2015 [22]. Corn flour (10.00 g) was extracted with absolute ethanol by shaking and filtering. The filtrate was titrated with a standard ethanolic potassium hydroxide solution, using phenolphthalein as an indicator. The free fatty acid content was expressed as the amount of potassium hydroxide (mg KOH) required to neutralize free fatty acids in 100 g of dry sample, calculated according to Equation (2):

(2) $$S=\frac{(V_{1}-V_{0})\times c\times 56.1\times (50/25)\times 100}{m\times (1-W/100)}$$

where *V*_1_ and *V*_0_ are the sample and blank titration volumes (mL), respectively; *c* is the KOH concentration (mol/L); *m* is the sample mass (g); and *W* is the moisture mass fraction of the sample, expressed as the mass of water contained in 100 g of sample (g). The constants 56.1, 50/25, and 100 account for the KOH molar mass, extraction-to-aliquot volume ratio, and mass conversion factor, respectively.

#### 2.5.2. Measurement of Corn Physiological Activity Indicators

##### Germination Rate

Germination rate was included as an indicator of physiological activity to assess whether physiological deterioration accompanied changes in nutritional quality and mycotoxin concentrations. Germination rates were determined according to the Chinese National Standard GB/T 5520-2011 [23]. For each condition, 100 intact and well-filled corn kernels (*n*_1_) were randomly selected from the total number of tested kernels. The kernels were soaked in distilled water at 25 °C for 6 h, evenly placed on germination trays, and incubated at 25 °C for 7 days. The trays were kept moist during incubation, and the number of germinated kernels (*n*_2_) was recorded. Germination rates were calculated using Equation (3).

(3) $$\mathrm{Germination rate}(\%)=\frac{n_{2}}{n_{1}}\times 100$$

##### Superoxide Dismutase Activity

A 0.10 g tissue sample was homogenized on ice in 1.0 mL of extraction buffer from a superoxide dismutase (SOD) assay kit (BC0170). The homogenate was centrifuged at 8000× *g* for 10 min at 4 °C. The supernatant was collected and placed on ice. The absorbance was measured at 560 nm with a spectrophotometer to determine SOD activity.

##### Catalase Activity

A 0.10 g tissue sample was homogenized on ice in 1.0 mL of extraction buffer from a catalase (CAT) activity assay kit (BC0200). The homogenate was centrifuged at 8000× *g* for 10 min at 4 °C. The supernatant was collected and placed on ice. The absorbance was measured at 240 nm with a spectrophotometer to determine CAT activity.

### 2.6. Statistical Analysis

Data are expressed as the mean ± standard deviation of three independent storage replicates per temperature at each sampling time. Each replicate value was obtained from a sample collected and analyzed separately from one storage unit. Statistical analyses were performed using IBM SPSS Statistics 27, and figures were generated using Origin 2024. Variance equality was assessed using Levene’s test, and normal distribution was checked using the Shapiro–Wilk test. Differences between temperature conditions at the same sampling time were analyzed using a one-way analysis of variance (ANOVA), followed by Duncan’s multiple-range test for post hoc comparison. Differences were considered statistically significant at *p* < 0.05 and highly significant at *p* < 0.01. A correlation analysis was performed using Origin 2024 to examine the relationships between the mycotoxins (FB_1_, FB_2_, FB_3_, DON, and ZEN) and quality parameters (crude protein, crude fat, starch, free fatty acids, germination rate, SOD activity, and CAT activity).

## 3. Results

### 3.1. Changes in Mycotoxin Concentrations During Storage

#### 3.1.1. Changes in Aflatoxins and OTA Concentrations

Among the mycotoxins analyzed, aflatoxins, including AFB_1_, AFB_2_, AFG_1_, AFG_2_, and ochratoxin A (OTA), were below the limit of detection in all corn samples throughout the 8-month storage period (Table 1). This is related to the fact that the initial levels of aflatoxins and OTA in the corn raw material were below the LOD.

#### 3.1.2. Changes in Fumonisin Concentrations (FB_1_, FB_2_, and FB_3_)

The changes in fumonisin concentrations in corn at different storage temperatures are shown in Figure 1. At 15 °C, the contents of FB_1_ and FB_2_ had decreased by 3.2% and 2.5%, respectively, by the eighth month compared to their initial values. FB_3_ reached 41.51 ± 2.10 μg/kg at month 8, representing a 17.3% decrease. At 35 °C, the three fumonisins increased by 13.8%, 40.0%, and 82.8%, respectively, compared with their initial concentrations. Overall, the three fumonisins remained relatively stable or showed a slow decline during storage at 15 °C, while they accumulated continuously at 35 °C. The difference between the two temperature conditions gradually widened with prolonged storage.

#### 3.1.3. Changes in DON Concentration

As shown in Figure 2, DON concentration showed distinct temperature-dependent changes during storage. In the 15 °C group, DON concentration remained nearly stable throughout the 8-month storage period. In contrast, DON concentration in the 35 °C group increased by 10.4% after eight months. The increase was more evident during the early to middle stages of storage and tended to slow after 6 months. These results indicate that 35 °C storage promoted DON accumulation, whereas 15 °C storage effectively maintained DON at a relatively stable level.

#### 3.1.4. Changes in Zearalenone (ZEN) Concentration

As shown in Figure 3, ZEN concentration remained relatively stable at 15 °C throughout storage. At 35 °C, ZEN concentration increased from the initial value to its highest (10.34 ± 0.10 μg/kg) observed level at month 6, followed by a decrease at months 7 and 8. Despite this subsequent decline, the concentration at month 8 remained approximately 26.1% higher than the initial concentration. Thus, ZEN showed a net increase over the eight-month storage period at 35 °C, although the increase was not continuous.

### 3.2. Fungal Community Structure and Succession

#### 3.2.1. Analysis of Fungal Alpha Diversity

The fungal alpha diversity was evaluated using multiple indices (Figure 4). Species richness was assessed using the Chao1 and observed species indices; community diversity was evaluated using the Shannon and Simpson indices; and community evenness was assessed using Pielou’s evenness index.

The 15 °C group showed slightly higher fungal richness than the 35 °C group, as reflected by the Chao1 index (*p* = 0.049) and the observed species index (*p* = 0.048). The Shannon index was also higher in the 15 °C group than in the 35 °C group (*p* = 0.036), indicating a modest reduction in fungal community diversity under 35 °C storage. By contrast, Good’s coverage (*p* = 0.37), Simpson index (*p* = 0.19), and Pielou’s evenness (*p* = 0.078) did not differ significantly between the two temperature groups, although Pielou’s evenness showed a decreasing tendency in the 35 °C group. Good’s coverage values were close to 1.0000 for both groups, confirming that the sequencing depth was sufficient to capture the major fungal taxa in the corn samples. Overall, high-temperature storage was associated with reduced fungal richness and Shannon diversity, whereas sequencing coverage, community dominance, and evenness remained broadly comparable between the two temperature conditions. These diversity indices do not quantify the total fungal load.

#### 3.2.2. Analysis of Fungal Beta Diversity

PCoA based on Bray–Curtis distances was performed to evaluate the differences in the fungal community structure between the two storage temperature groups. As shown in Figure 5, PC1 and PC2 explained 35.2% and 26.2% of the total variation, respectively, and accounted for 61.4% of the cumulative variation. The samples from the 15 °C and 35 °C groups partially overlapped in the ordination space, indicating that the overall fungal community structures were not clearly separated between the two temperature conditions. However, several samples from the 35 °C group shifted toward the positive direction of PC2, whereas samples from the 15 °C group were mainly distributed in the negative or central region of PC2. These results suggest that high-temperature storage may have influenced fungal community composition to some extent, although the overall similarity between the two groups remained relatively high.

#### 3.2.3. Fungal Community Composition

At the phylum level, Ascomycota dominated all samples, with relative abundances ranging from 0.55 to 0.99 (Figure 6A). No obvious differences in phylum-level composition were observed among the various temperature conditions, indicating that the storage temperature did not cause marked restructuring of the fungal community at the phylum level.

At the genus level, *Fusarium* and *Trichoderma* showed the most evident responses to the storage temperature (Figure 6B). *Fusarium* showed higher relative abundance at 35 °C (0.42–0.73). *Trichoderma* was more abundant at 15 °C and remained scarce at 35 °C. *Sarocladium* was also a prominent component of the communities and varied over time in both conditions.

At the species level, *Fusarium proliferatum* and *Fusarium culmorum* were the dominant species within the genus *Fusarium*, whereas the dominant species of *Trichoderma* and *Sarocladium* were *Trichoderma aggressivum* and *Sarocladium kiliense*, respectively (Figure 6C). At 35 °C, their relative contributions shifted over time, with *F. culmorum* becoming more prominent during months 3–4 and *F. proliferatum* increasing again at month 5. *Trichoderma aggressivum* and *Sarocladium kiliense* showed temporal peaks in relative abundance at 15 °C.

### 3.3. Changes in Corn Quality Indicators During Storage

#### 3.3.1. Changes in Corn Nutritional Quality Under Different Storage Temperatures

As shown in Figure 7A, the crude protein content changed only slightly during the 8-month storage period in both temperature groups. These results suggest that crude protein content was only weakly affected by storage temperature. Crude fat declined more at 35 °C than at 15 °C (Figure 7B). Starch content in the 35°C group decreased by 10.9% from the initial value, showing a greater reduction than that observed in the 15 °C group (Figure 7C). Free fatty acids increased with storage time in both groups, but the increase was more pronounced at 35 °C (Figure 7D). Under high-temperature storage conditions, lipid hydrolysis and oxidation may have promoted the accumulation of free fatty acids, leading to a sharp increase in free fatty acids.

#### 3.3.2. Physiological Activity Indicators

As shown in Figure 8A, the germination rate decreased during storage, with a faster decline in the 35 °C group. After eight months of storage, the germination rate declined from approximately 97% to 72% in the 15 °C group, whereas it dropped to 9% in the 35 °C group, indicating that prolonged high-temperature storage markedly reduced kernel viability. SOD and CAT activities also declined during storage at both temperatures, with sharper decreases at 35 °C (Figure 8B,C). The decline in antioxidant enzyme activity generally followed the same trend as that in the germination rate.

### 3.4. Associations Between Mycotoxin Concentrations and Corn Quality Indicators

#### 3.4.1. Correlation Between Mycotoxins and Quality Indicators

At 15 °C, FB_1_, FB_2_, and FB_3_ were highly positively correlated with each other, and DON was also highly positively correlated with ZEN. Overall, fumonisins were negatively correlated with DON and ZEN, and some pairwise correlations were statistically significant. Overall, the three fumonisins tended to be positively correlated with nutritional components, germination rate, and antioxidant enzyme activities and negatively correlated with free fatty acids; however, not all correlations were statistically significant. DON and ZEN generally showed negative correlations with most quality indicators, with significant negative correlations observed between ZEN and crude fat content and between ZEN and SOD activity. Starch, crude fat, and crude protein contents were positively correlated with each other at significant or highly significant levels. Free fatty acid content was negatively correlated with nutritional components, germination rate, and antioxidant enzyme activity, whereas germination rate, SOD activity, and CAT activity were highly positively correlated with each other (Figure 9A).

At 35 °C, all mycotoxins were significantly positively correlated with each other, with most correlations reaching *p* ≤ 0.001. FB_1_, FB_2_, FB_3_, and DON showed highly significant negative correlations with starch, crude fat, crude protein, germination rate, SOD activity, and CAT activity but were significantly positively correlated with free fatty acids. ZEN showed the same correlation direction as these quality indicators, although its correlation strength was relatively weaker. Starch, crude fat, and crude protein contents were highly positively correlated with each other, whereas free fatty acids were highly negatively correlated with germination rate, SOD activity, and CAT activity. The germination rate was highly positively correlated with SOD and CAT activities. These correlation patterns among the non-mycotoxin indicators were consistent with those observed during low-temperature storage at 15 °C (Figure 9B).

#### 3.4.2. Exploratory Multiple Linear Regression Analysis

Regression equations were fitted separately for each detected mycotoxin based on the existing dataset. As shown in Table 2, these equations summarize the joint associations between mycotoxin concentrations and quality indicators in the observed dataset. At 15 °C, different mycotoxins exhibited varying correlations with nutritional parameters. At 35 °C, the coefficients for crude fat were all negative in the equations for the five mycotoxins, while the coefficients for crude protein were generally positive. Crude fat is the quality parameter that correlates in the same direction as changes in mycotoxin levels at this temperature. The analysis of the regression equation was conducted to further elucidate the correlation between mycotoxin production and quality indicators, complementing the findings of previous studies.

## 4. Discussion

### 4.1. The Effect of Storage Temperature on Changes in Mycotoxins in Corn

Under a constant relative humidity of 65 ± 5%, storage at 15 °C and 35 °C resulted in clearly distinct mycotoxin dynamics. Under low-temperature storage at 15 °C with constant relative humidity, FBs, DON, and ZEN remained stable or decreased slightly, with no obvious risk of accumulation. This finding is consistent with the results of Shi et al. [24], who pointed out that mycotoxin concentrations showed a temperature-dependent gradient under different storage conditions, confirming the inhibitory effect of low temperature on toxin accumulation. The high-temperature, constant-humidity condition at 35 °C served as a key driver of mycotoxin accumulation, under which detected mycotoxins generally showed a continuously increasing trend. This observation is consistent with the findings of Mannaa et al. [25]. In the present study, the continuous accumulation of FBs at 35 °C may reflect the activation of the fumonisin biosynthetic gene cluster (*FUM* cluster) under high-temperature storage, which promoted toxin biosynthesis. At 35 °C, the ZEN concentration peaked in the sixth month of storage and then declined, possibly due to its conversion into a hidden mycotoxin. Tan et al. reported that ZEN can bind to maize alcohol-soluble proteins through noncovalent interactions, forming hidden ZEN that is not detected by conventional analytical methods [26].

### 4.2. Analysis of Fungal Communities During Corn Storage

Results suggest that changes in mycotoxin concentrations during corn storage are associated with shifts in fungal community composition. High-temperature storage was associated with reduced fungal richness and Shannon diversity. Ascomycota remained the dominant phylum throughout storage. However, clear temperature-related differences were observed at the genus level, particularly for *Fusarium* and *Trichoderma*. The inhibitory effects of *Trichoderma* against *Fusarium* through nutrient and space competition, mycoparasitism, and the production of cell wall-degrading enzymes have been reported previously [27,28]. These results suggest that the storage temperature may affect mycotoxin dynamics in corn. These findings provide a theoretical basis for optimizing corn storage conditions and controlling mycotoxin contamination.

### 4.3. Analysis of Mycotoxins and Corn Quality During Storage

In the 35 °C group, the decrease in total starch content may be related to the elevated respiration rate of corn kernels during deterioration. Higher respiratory activity may require greater starch degradation [29]. Crude protein content was only weakly affected by storage temperature. Timm et al. reported that the soluble protein content tended to decrease with increasing storage time at storage temperatures of 15 °C and 25 °C [30]. Previous studies have shown that a high proportion of moldy kernels in corn can directly increase free fatty acid content and is accompanied by elevated mycotoxin concentrations [31]. In the present study, free fatty acids increased to approximately 45 mg KOH/100 g under the 35 °C condition, showing a synchronous upward trend with the continuous increase in fumonisin concentrations at high temperatures. This suggests that high-temperature storage may simultaneously promote lipid hydrolysis and fungal toxin production. As a core indicator of corn storage quality, free fatty acid content may serve as a useful supplementary indicator for assessing storage deterioration and potential mycotoxin risk. The greater decline in germination at 35 °C occurred together with decreases in SOD and CAT activities, suggesting that physiological deterioration accompanied the changes in nutritional composition. Therefore, there is a correlation between germination rate and SOD and CAT levels. Germination rate, SOD, and CAT were included as indicators of physiological activity to assess whether physiological deterioration accompanied changes in nutritional quality and mycotoxin concentrations. Xue et al. [13] showed that *Fusarium* infection led to pronounced kernel darkening and a substantial increase in the number of surface fungal spores. Infection with *Fusarium verticillioides* was also associated with elevated malondialdehyde levels, ultimately accelerating the deterioration of kernel quality. Satya and Nagarajappa [32] investigated the mechanisms of seed deterioration during the artificial aging of corn and found that as aging time increased, SOD and CAT activities decreased, leading to reductions in the germination rate and vigor index.

Correlation analysis further showed that the relationships between mycotoxins and quality indicators differed markedly under different temperature conditions. Crude protein, crude fat, and starch characterized nitrogen-containing constituents, lipid reserves, and carbohydrate reserves, respectively. Free fatty acid content provided an indicator of lipid hydrolysis, while germination rate reflected kernel viability. SOD and CAT activities characterized antioxidant enzyme activity. Taylor et al. [33] further pointed out that the activities of lipases and oxidases during corn storage directly determine the rate of increase in free fatty acids. Under the 35 °C condition, the simultaneous increase in several mycotoxins and the concurrent decreases in nutritional components, germination rate, and antioxidant enzyme activities led to relatively strong negative correlations between mycotoxins and these quality indicators. At 15 °C, changes in both mycotoxins and most nutritional indicators were limited. Mycotoxin accumulation may further aggravate kernel quality deterioration, potentially through oxidative stress, disruption of cellular metabolism, or membrane damage. Therefore, there may be a bidirectional interaction between quality deterioration and mycotoxin accumulation.

### 4.4. Limitations and Future Research

The regression equation was calculated based on existing data and requires further validation. Furthermore, no interventional treatments aimed at protecting kernel quality or blocking toxin biosynthesis were included in this study, and the proposed bidirectional causal relationship needs to be verified through controlled experiments that inhibit quality deterioration or block mycotoxin biosynthesis. Total fungal load was not independently quantified, and toxin biosynthetic gene expression, toxin degradation, fungal antagonism, and oxidative damage were not directly assessed.

Future work that combines quantitative fungal measurements with targeted mechanistic and intervention experiments would help distinguish shared responses to storage conditions from causal relationships between quality deterioration and mycotoxin accumulation. In practical applications, additional independent observational data should be used to evaluate the stability and potential predictive value of the regression model.

## 5. Conclusions

Over eight months of storage, naturally contaminated corn showed different patterns of mycotoxin accumulation, fungal community composition, and quality deterioration at 15 and 35 °C. At 15 °C, FB_1_, FB_2_, DON, and ZEN remained stable, while FB_3_ showed an overall decrease. At 35 °C, FB_1_, FB_2_, FB_3_, DON, and ZEN accumulated continuously. Although PCoA showed no pronounced overall separation between the two temperature conditions, alpha diversity analysis indicated lower fungal richness and Shannon diversity at 35 °C. The 35 °C group also showed a higher relative abundance of *Fusarium* and a lower relative abundance of *Trichoderma*, accompanied by more severe starch and crude fat losses, increased free fatty acids, reduced germination rate, and decreased antioxidant enzyme activities. Under the storage conditions used in this study, maintaining the temperature at 15 °C markedly slowed mycotoxin accumulation and quality deterioration. Free fatty acids, germination rates, and SOD and CAT activities may serve as supplementary indicators of the storage status; however, they cannot replace the direct detection of mycotoxins. These findings provide experimental support for optimizing long-term corn storage conditions and establishing multi-indicator risk-monitoring strategies.

## Figures and Tables

**Figure 1:**
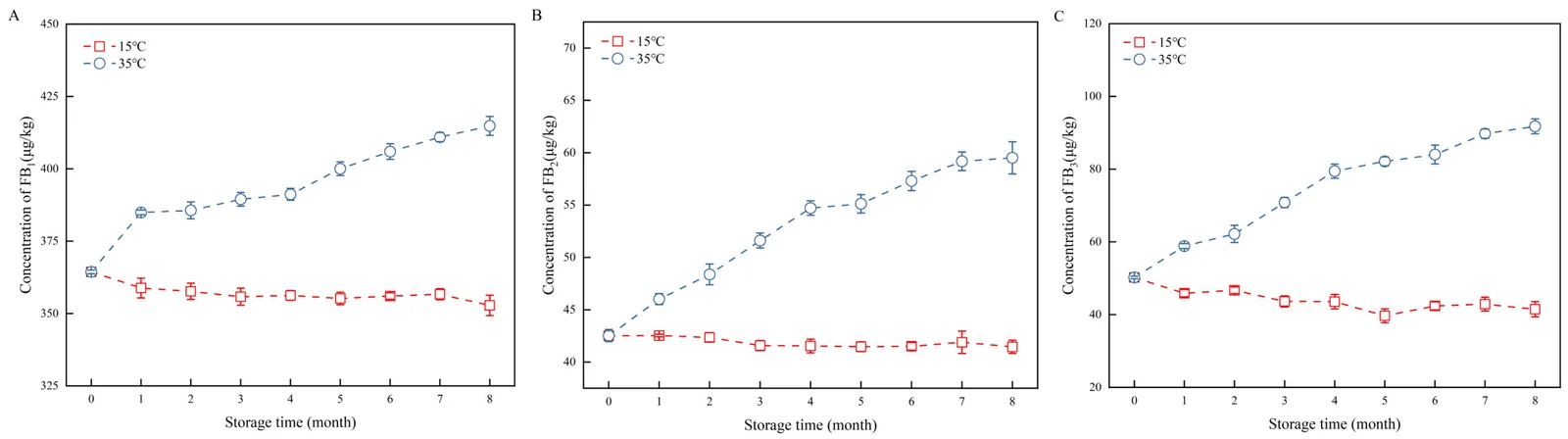
Changes in fumonisin concentrations in corn during storage at 15 and 35 °C: (**A**) Fumonisin B_1_ (FB_1_); (**B**) Fumonisin B_2_ (FB_2_); (**C**) Fumonisin B_3_ (FB_3_). Data are presented as mean ± standard deviation (*n* = 3).

**Figure 2:**
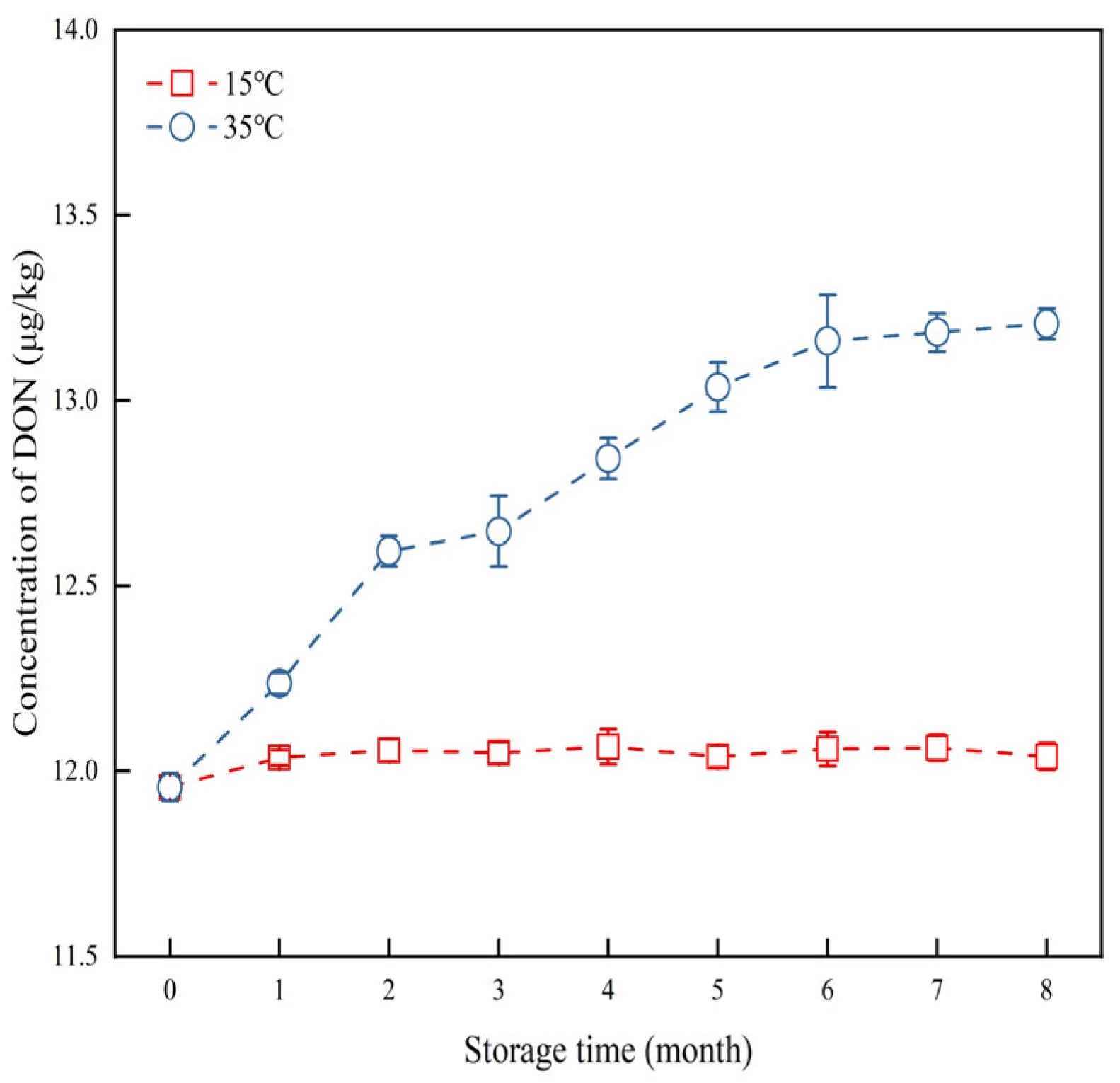
Changes in DON concentration in corn during storage at 15 and 35 °C. Data are presented as mean ± standard deviation (*n* = 3).

**Figure 3:**
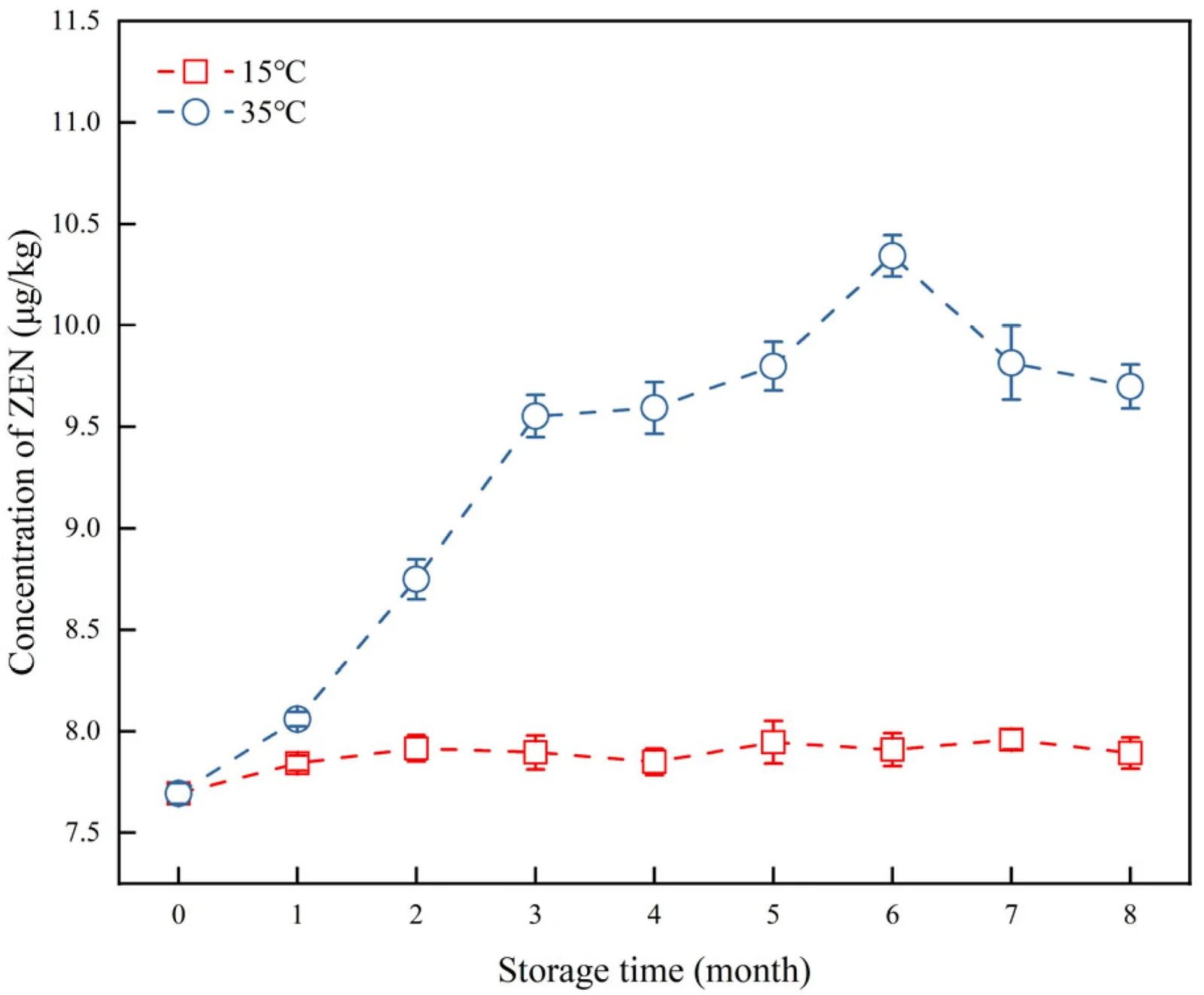
Changes in ZEN concentration in corn during storage at 15 and 35 °C. Data are presented as mean ± standard deviation (*n* = 3).

**Figure 4:**
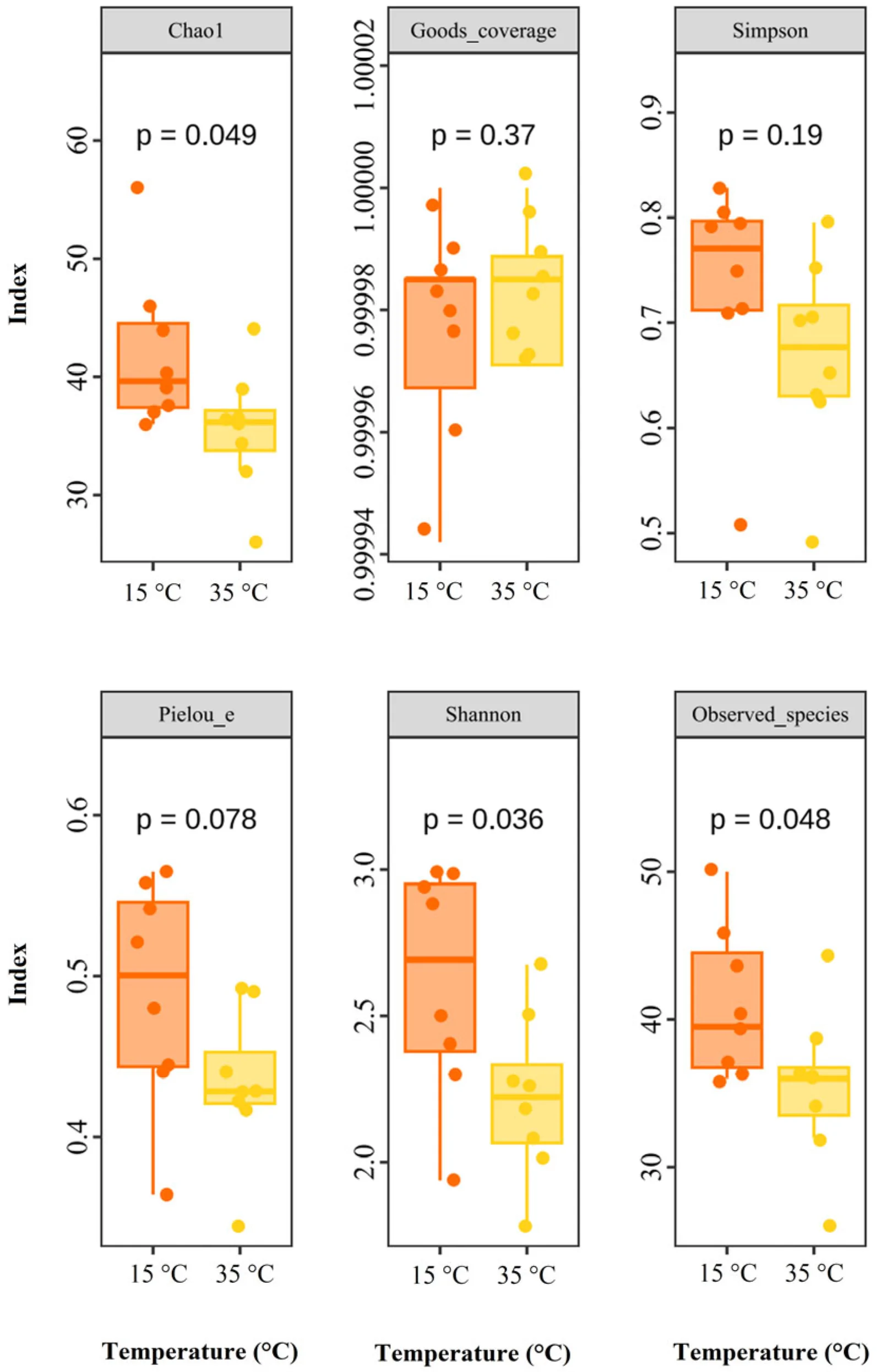
Boxplots of fungal alpha-diversity indices under different storage temperatures. The center line indicates the median, boxes represent the interquartile range, and whiskers indicate the data range. *p* values are shown for comparisons between groups.

**Figure 5:**
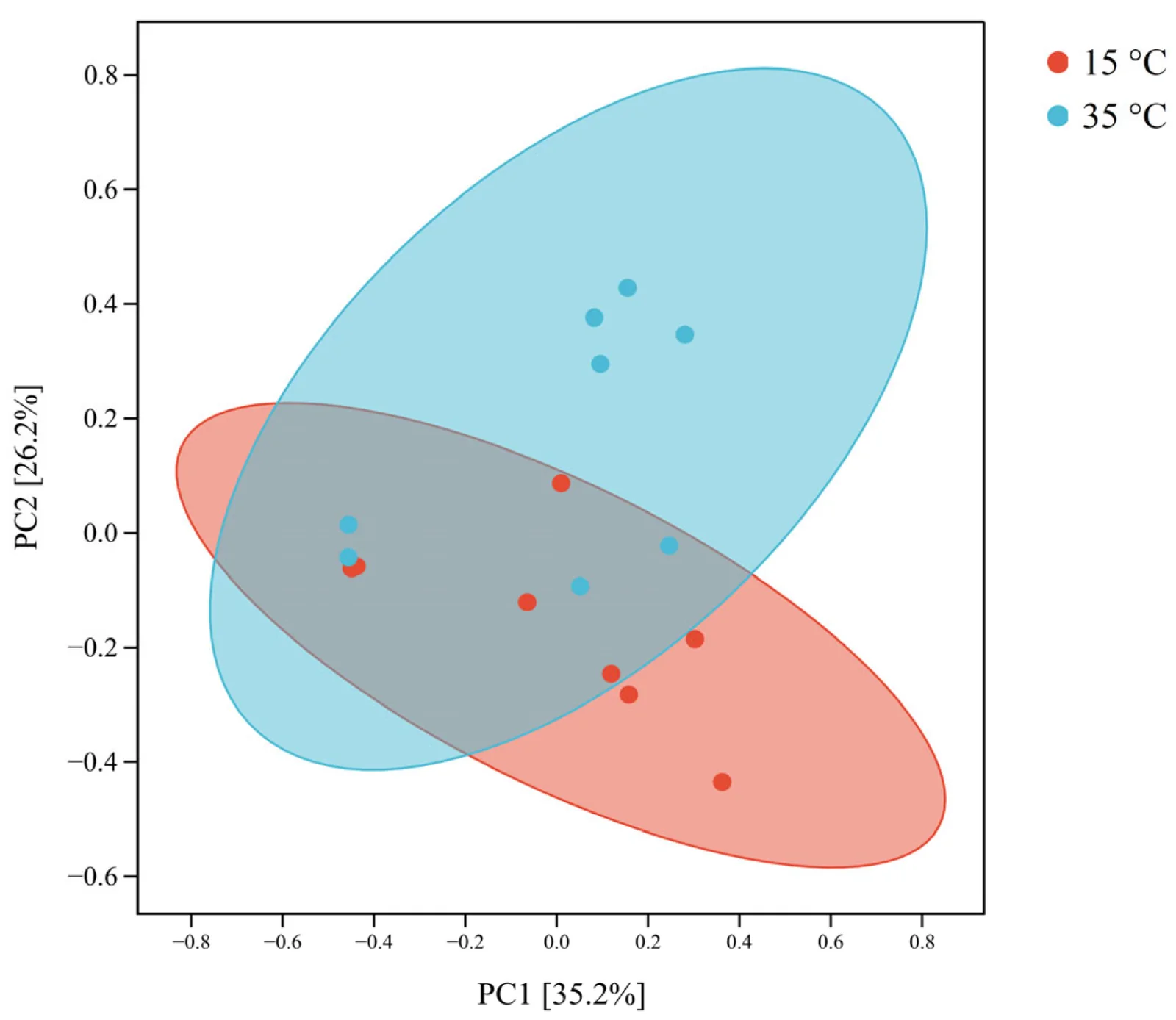
PCoA of fungal communities in corn based on Bray–Curtis dissimilarities.

**Figure 6:**
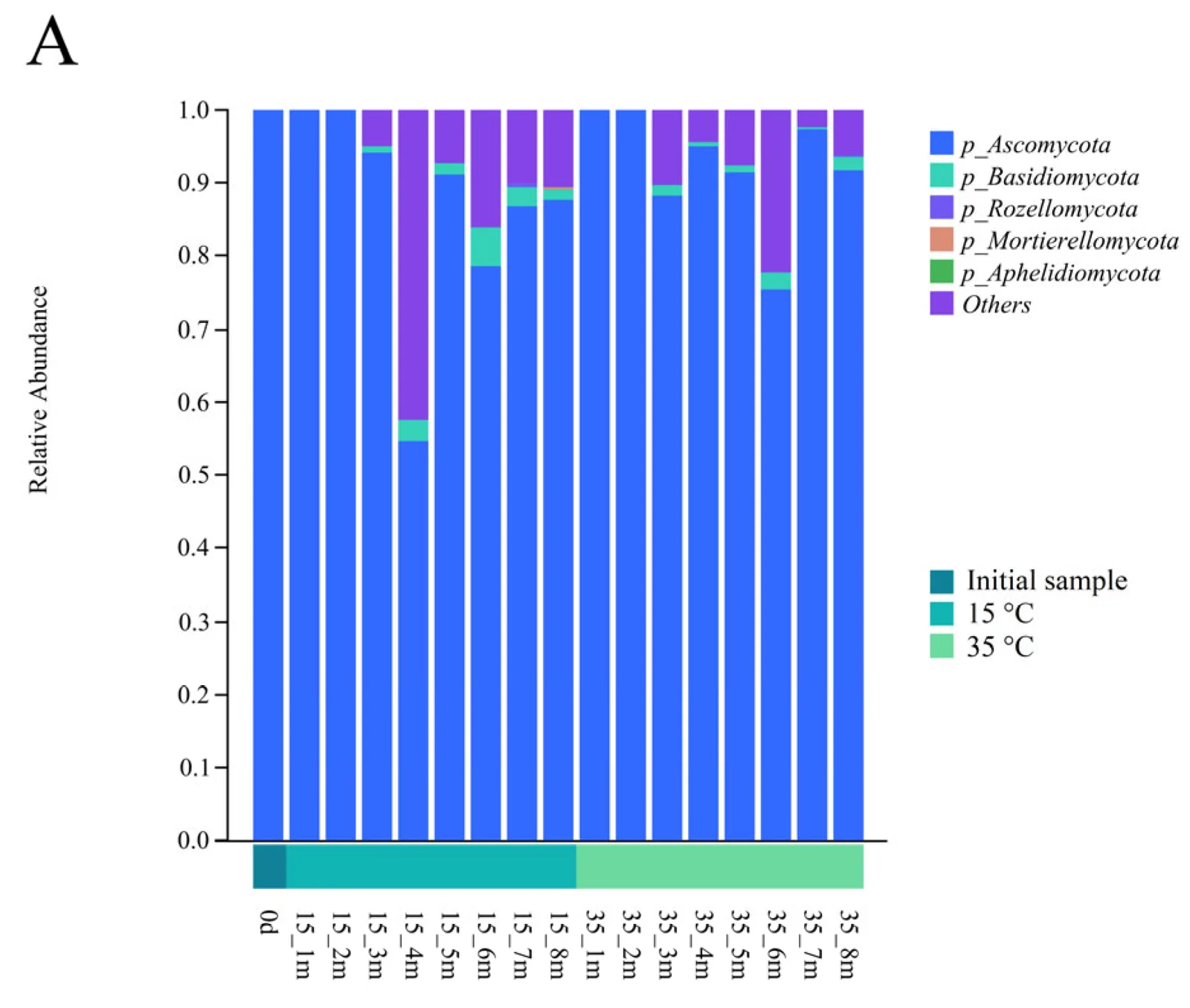

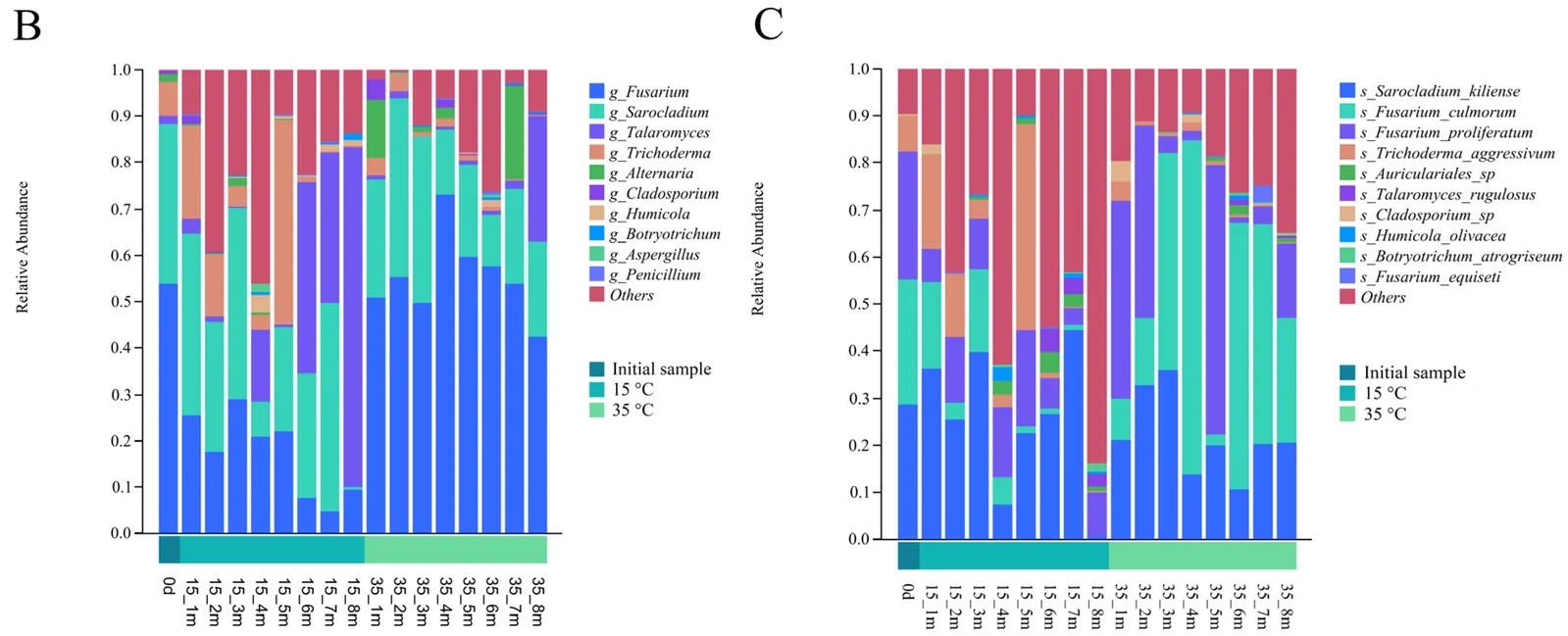
Relative abundance of fungal communities in corn during storage at different temperatures: (**A**) phylum level; (**B**) genus level; (**C**) species level.

**Figure 7:**
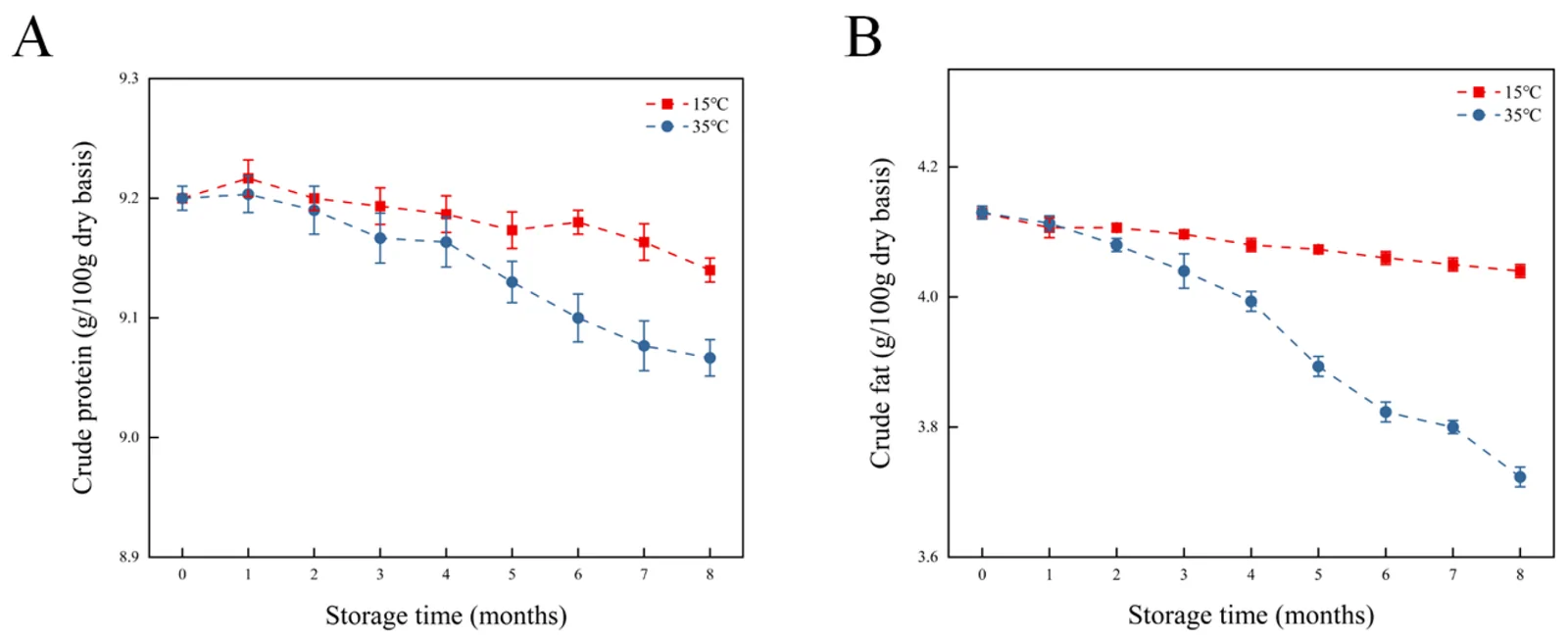

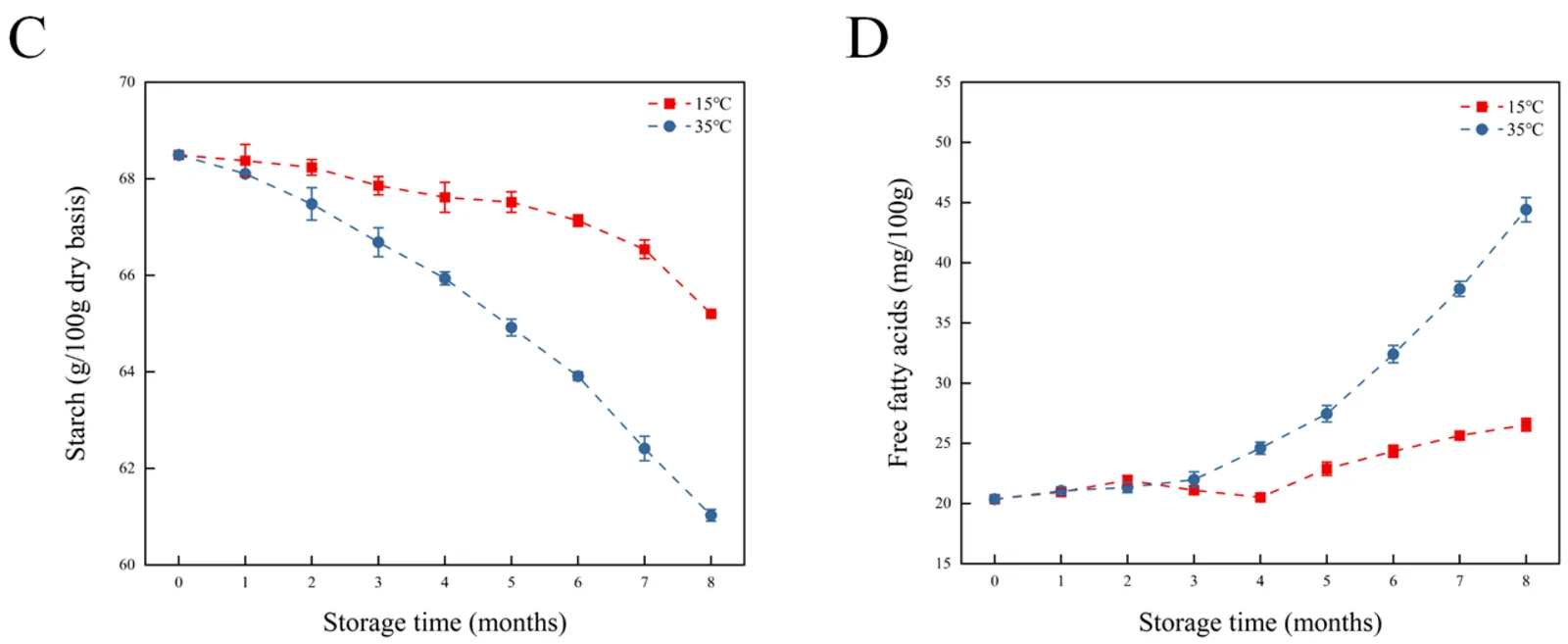
Changes in corn nutritional quality and free fatty acids during storage: (**A**) crude protein content; (**B**) crude fat content; (**C**) starch content; (**D**) free fatty acid content. Data are presented as mean ± standard deviation (*n* = 3).

**Figure 8:**
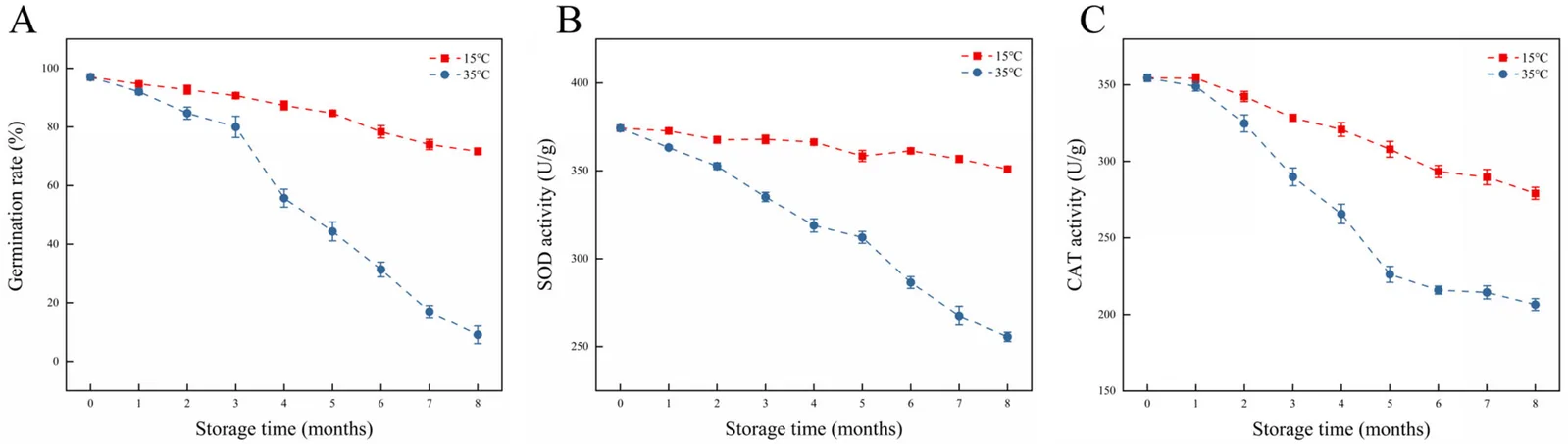
Changes in corn physiological activity indicators during storage: (**A**) germination rate; (**B**) SOD activity; (**C**) CAT activity. Data are presented as mean ± standard deviation (*n* = 3).

**Figure 9:**
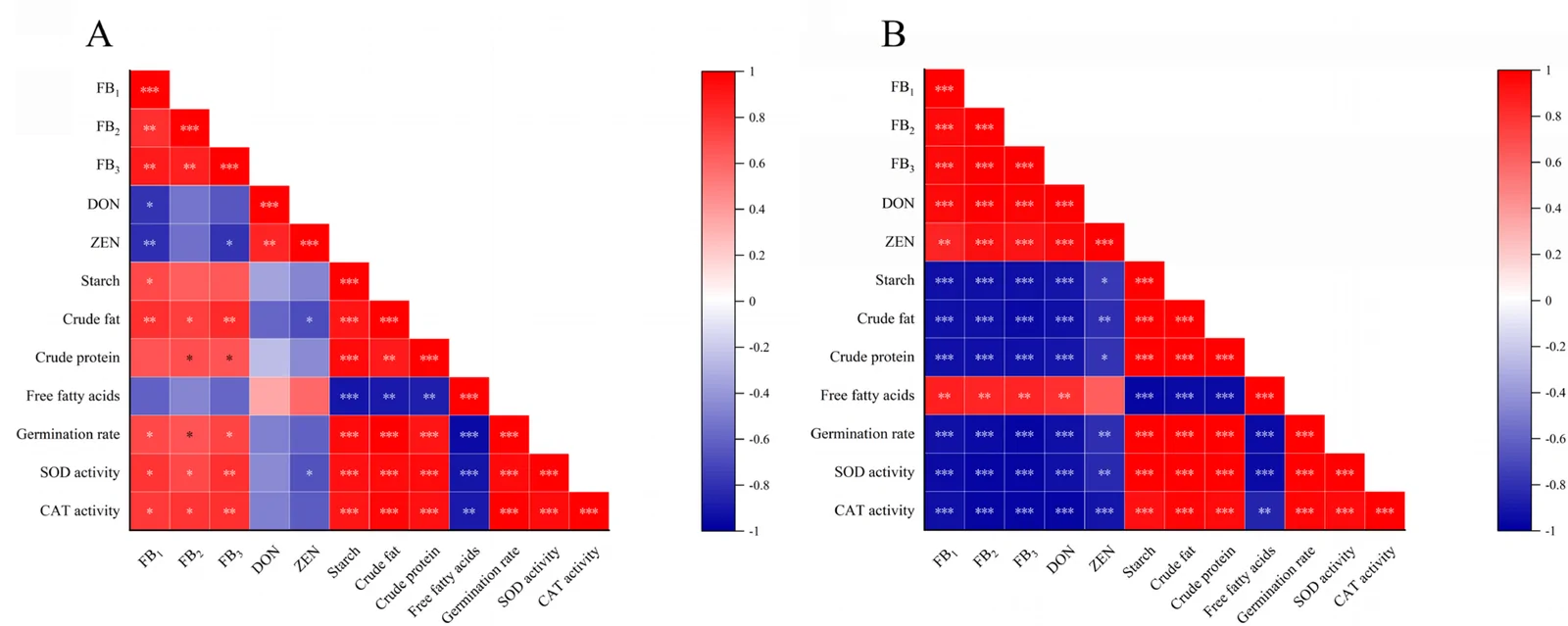
Correlations among mycotoxins and corn quality indicators under different storage temperatures: (**A**) 15 °C storage group; (**B**) 35 °C storage group. The color scale represents correlation coefficients, with red indicating positive correlations and blue indicating negative correlations. *, *p* < 0.05; **, *p* < 0.01; ***, *p* < 0.001.

**Table 1:** Detection results of aflatoxins and OTA content during storage from 0 to 8 months.

Mycotoxin	15 °C (0–8 Months)	35 °C (0–8 Months)
AFB_1_	<LOD	<LOD
AFB_2_	<LOD	<LOD
AFG_1_	<LOD	<LOD
AFG_2_	<LOD	<LOD
OTA	<LOD	<LOD

**Table 2:** Exploratory regression equations linking mycotoxins and quality indices at different storage temperatures.

Storage Temperature	Mycotoxin	Regression Equation
15 °C	FB_1_	FB_1_= −973.25X_1_ − 788.22X_2_ + 11.81X_3_ + 5.94X_4_ + 2.10X_5_ + 3.01X_6_ + 0.21X_7_ + 10,240.78
	FB_2_	FB_2_= −28.64X_1_ − 15.53X_2_ + 0.54X_3_ + 0.24X_4_ − 0.08X_5_ + 0.07X_6_ + 0.06X_7_ + 291.02
	FB_3_	FB_3_= 286.62X_1_ + 633.56X_2_ − 3.46X_3_ − 2.90X_4_ − 2.56X_5_ − 0.81X_6_ + 0.16X_7_ − 4410.62
	DON	DON= 20.15X_1_ + 20.17X_2_ − 0.18X_3_ − 0.15X_4_ − 0.08X_5_ − 0.06X_6_ + 0.00X_7_ − 210.19
	ZEN	ZEN= 33.48X_1_ + 33.30X_2_ − 0.24X_3_ − 0.22X_4_ − 0.12X_5_ − 0.12X_6_ − 0.00X_7_ − 362.00
35 °C	FB_1_	FB_1_= 212.89X_1_ − 592.14X_2_ − 33.34X_3_ − 12.24X_4_ − 0.40X_5_ − 0.35X_6_ + 1.31X_7_ + 3090.42
	FB_2_	FB_2_= 61.98X_1_ − 30.25X_2_ − 3.44X_3_ − 1.24X_4_ − 0.05X_5_ − 0.19X_6_ + 0.06X_7_ − 89.67
	FB_3_	FB_3_= 194.58X_1_ − 1.04X_2_ − 7.36X_3_ − 1.53X_4_ − 0.13X_5_ − 0.26X_6_ − 0.05X_7_ − 1074.60
	DON	DON= 4.50X_1_ − 12.59X_2_ − 0.84X_3_ − 0.31X_4_ − 0.02X_5_ − 0.01X_6_ + 0.03X_7_ + 80.01
	ZEN	ZEN= 7.75X_1_ − 11.86X_2_ − 0.03X_3_ − 0.21X_4_ + 0.03X_5_ − 0.05X_6_ + 0.00X_7_ + 7.72

Note: X_1_, crude protein content (g/100 g); X_2_, crude fat content (g/100 g); X_3_, starch content (g/100 g); X_4_, free fatty acids (mg KOH/100 g); X_5_, germination rate (%); X_6_, SOD activity (U/g); X_7_, CAT activity (U/g).

## Data Availability

The original contributions presented in this study are included in the article/Supplementary Materials. Further inquiries can be directed to the corresponding authors.

## Supplementary Materials

The following supporting information can be downloaded at: https://www.mdpi.com/article/10.3390/foods15193438/s1, Table S1: Mobile phase gradient; Table S2: Mass spectrometry parameters for the ten mycotoxins; Table S3: Key performance characteristics of detection methods for the ten mycotoxins.

## Abbreviations

The following abbreviations are used in this manuscript:

ANOVA	Analysis of variance
AFs	Aflatoxins
AFB_1_	Aflatoxin B_1_
AFB_2_	Aflatoxin B_2_
AFG_1_	Aflatoxin G_1_
AFG_2_	Aflatoxin G_2_
ASV	Amplicon sequence variant
CAT	Catalase
DON	Deoxynivalenol
ESI	Electrospray ionization
FBs	Fumonisins
FB_1_	Fumonisin B_1_
FB_2_	Fumonisin B_2_
FB_3_	Fumonisin B_3_
*F. proliferatum*	*Fusarium proliferatum*
*F. culmorum*	*Fusarium culmorum*
*F. graminearum*	*Fusarium graminearum*
HPLC–MS/MS	High-performance liquid chromatography–tandem mass spectrometry
LC–MS/MS	Liquid chromatography–tandem mass spectrometry
MDPI	Multidisciplinary Digital Publishing Institute
MRM	Multiple reaction monitoring
OTA	Ochratoxin A
PCoA	Principal coordinates analysis
*S. kiliense*	*Sarocladium kiliense*
SOD	Superoxide dismutase
*T. aggressivum*	*Trichoderma aggressivum*
ZEN	Zearalenone
