# Supplementary material for: Dynamics of Mycotoxins, Microbial Communities, and Quality of Corn When Stored at Various Temperatures
Source: Foods. 2026 Sep 25;15(19):3438. doi: 10.3390/foods15193438 (PMC13664013; doi:10.3390/foods15193438)
Supplement: Supplementary file 1 [file foods-15-03438-s001.zip › foods-4533499-supplementary.pdf]

Tables in Supplementary Materials

Table S1. Mobile phase gradient.

Time (min)	A (%)	B (%)
0.0	95	5
2.0	95	5
10.0	5	95
11.5	5	95
12.0	95	5
17.0	95	5

Table S2. Mass spectrometry parameters for the ten mycotoxins.

Mycotoxins	Quantitative Ions (m/z)	Qualitative Ions (m/z)	Collision Energy (V)	Declustering Potential (V)	Retention Times (ms)
AFB_1	313	241	50	103	60
		285	31	25	60
AFB_2	315	287	36	103	60
		259	40	98	60
AFG_1	329	243	38	26	60
		283	36	27	60
AFG_2	331	245	29	100	60
		285	38	100	60
FB_1	722	352	50	100	60
		334	50	100	60
FB_2	706	336	50	100	60
		354	42	100	60
FB_3	706	336	50	100	60
		354	42	100	60
OTA	404	239	32	85	60
		358	20	85	60
DON	296.9	249.1	12	80	60
		231.1	18	80	60
ZEN	317.1	131	-40	-80	60
		175	-34	-80	60
		273	-26	-80	60

Table S3. Key performance characteristics of detection methods for the ten mycotoxins [1].

Mycotoxins	Linear Range (μg/mL)	Linear Equation	R^2	LOD (μg/kg)	LOQ (μg/kg)	Recovery (%)
AFB_1	0.001-0.2	y = 26875.42401x	0.9951	0.0993	0.2980	83-102
AFB_2	0.001-0.2	y = 7264.58912x	0.9985	0.1139	0.3418	82-102
AFG_1	0.001-0.2	y = 4945.30291x	0.9995	0.0805	0.2414	84-98
AFG_2	0.001-0.2	y = 3002.35794x	0.9979	0.8017	2.4051	83-96
FB_1	0.002-0.5	y = 6262.06910x	0.9985	1.9570	3.1615	85-91
FB_2	0.002-0.5	y = 2790.41347x	0.9957	1.2552	1.8509	82-92
FB_3	0.002-0.5	y = 3754.20592x	0.9996	1.4412	4.8189	83-89
OTA	0.001-0.2	y = 71324.42701x	0.9987	0.1178	0.3928	86-93
DON	0.001-0.2	y = 595.26060x	0.9999	3.6571	9.1905	87-100
ZEN	0.001-0.2	y = 11750.67788x	0.9999	0.5834	1.9448	84-95

Note: The method validation data are described in our previous report [1].

Reference

1. Lyu, P.; Yuan, Y.; Chen, Y.; Liu, J.; Li, X.; Cai, D. Determination and contamination analysis of mycotoxins in corn by high-performance liquid chromatography-tandem mass spectrometry. *J. Maize Sci.* **2026**, 1–13. (In Chinese)
